# Waist-to-height ratio and coronary artery calcium incidence: the Brazilian Longitudinal Study of Adult Health (ELSA-Brasil)

**DOI:** 10.1016/j.lana.2025.101281

**Published:** 2025-10-31

**Authors:** Thiago Bosco Mendes, Giuliano Generoso, Ronaldo C. Fabiano, Bruno Halpern, Carolina Castro Porto Silva Janovsky, Carlos Manuel Romero, Raul D. Santos, Isabela Bensenor, Paulo Andrade Lotufo, Marcio Sommer Bittencourt

**Affiliations:** a Division of Endocrinology, Department of Medicine, University of Pittsburgh Medical Center, Pittsburgh, USA; b Center for Clinical and Epidemiological Research, University of São Paulo University Hospital, São Paulo, Brazil; c Department of Medicine, Brigham and Women's Hospital, Harvard Medical School, Boston, USA; d Obesity Center, 9 de Julho Hospital, São Paulo, Brazil; e Brazilian Association for the Study of Obesity (ABESO), São Paulo, Brazil; f Division of Endocrinology, Federal University of São Paulo, São Paulo, Brazil; g Division of Cardiology, University of Pittsburgh Medical Center, Pittsburgh, USA; h Heart Institute (InCor), University of Sao Paulo Medical School Hospital, São Paulo, Brazil; i Division of Cardiology, Department of Medicine, University of Pittsburgh, Pittsburgh, USA

**Keywords:** Coronary artery calcium, Subclinical atherosclerosis, Waist-to-height ratio, WHtR, Waist circumference, Body mass index, BMI, Obesity

## Abstract

**Background:**

Obesity is a cardiovascular risk factor and coronary artery calcium (CAC) is frequently used to assess coronary atherosclerosis burden. The purpose of this study was to evaluate body mass index (BMI), waist circumference (WC), and waist-to-height ratio (WHtR) as predictors of CAC incidence.

**Methods:**

We analyzed ELSA-Brasil cohort participants with no cardiovascular disease who had an initial CAC score of zero and repeated the test. Multivariate logistic regression analyses were performed to assess BMI, WC, and WHtR as predictors of CAC incidence.

**Findings:**

A total of 2721 participants (mean age 48.1 ± 7.56 years, 62.6% females) self-reported as White (57%), Brown/mixed (22.8%), Black (15.4%), Asian (4%) or Native/Indigenous (0.9%) were analyzed. CAC incidence after a mean of 5.24 years was 15.5% (confidence interval [CI] 95%: 14.2–17%). In unadjusted analysis, BMI, WC, and WHtR were positively associated with CAC incidence with an odds ratio (OR) of 1.19 (CI 95%: 1.08–1.31), 1.37 (CI 95% 1.23–1.52) and 1.39 (CI 95%: 1.25–1.54) per standard deviation, respectively. In the fully adjusted model, WHtR was the only independent predictor of CAC incidence, OR: 1.18 (CI 95% 1.03–1.35) per standard deviation. This effect was mainly driven by individuals with BMI <30 kg/m^2^.

**Interpretation:**

WHtR was the only independent anthropometric measure predictor of atherosclerosis incidence assessed by coronary artery calcium score. This effect is particularly relevant in individuals with BMI <30 kg/m^2^.

**Funding:**

10.13039/501100003593National Council for Scientific and Technological Development (CNPq), Brazil.

**Research in context**

**Evidence before this study**

Obesity is a complex metabolic disease that is usually assessed by body mass index (BMI) but can also be assessed by waist circumference (WC) and waist-to-height ratio (WHtR). We conducted a literature review on PubMed using the terms “Coronary artery calcium score”, “CAC”, “WHtR”, “waist-to-height-ratio”, “WC”, “waist circumference”, “BMI”, and “body mass index”. Previous studies have shown that WC and WHtR overperform BMI in identifying individuals with cardiometabolic risk factors and that BMI, WC, and WHtR are associated with prevalence of coronary artery calcium (CAC), a proxy of coronary atherosclerosis.

**Added value of this study**

Among individuals with a baseline CAC score of zero who underwent repeat testing after a mean of 5.24 years, BMI, WC, and WHtR were associated with incident CAC in unadjusted analyses. However, after adjustment for all classic cardiovascular risk factors, only WHtR remained significantly associated with CAC incidence. Upon stratification by BMI, it was found that WHtR association with CAC incidence is mainly driven by individuals with BMI <30 kg/m^2^.

**Implications of all the available evidence**

Increased central obesity measured as WHtR is associated with atherosclerosis development even after adjustment for classic cardiovascular risk factors, a finding especially relevant in individuals with BMI <30 kg/m^2^, which is below the classic obesity range.

## Introduction

Obesity is a complex metabolic disease defined by the World Health Organization as excessive fat deposition resulting in impaired health outcomes,^1^ including a higher incidence of cardiovascular disease (CVD).^2^, ^3^, ^4^, ^5^, ^6^, ^7^, ^8^, ^9^, ^10^, ^11^, ^12^, ^13^, ^14^, ^15^, ^16^, ^17^ Body mass index (BMI) has recently been incorporated into the PREVENT risk score equation as a predictor of CVD^18^ and is often used to delineate obesity given its simplicity and practical convenience.^19^

A BMI ≥30 kg/m^2^ is widely accepted as the cutoff to define obesity,^19^ but it is known that there is significant variability in fat tissue content and fat mass deposition site for any given BMI.^19^^,^^20^ Visceral adiposity is the one associated with pro-atherogenic lipid profile including low HDL-cholesterol, high triglycerides, increased ApoB-to-LDL cholesterol, and small dense LDL-cholesterol, as well as a proinflammatory state^21^, ^22^, ^23^; on the other side, subcutaneous fat has been shown to be cardioprotective.^24^ As such, visceral, or central fat, has a stronger association with adverse metabolic effects such as insulin resistance, dyslipidemia, and type 2 diabetes.^19^ Additionally, waist-to-height-ratio (WHtR) performs superiorly than BMI^25^, ^26^, ^27^, ^28^ and WC^28^, ^29^, ^30^ in identifying those at higher risk for cardiometabolic outcomes. Accordingly, different groups have recently recommended using anthropometric measures beyond BMI in clinical practice. The European Association for Study of Obesity (EASO) proposed a new framework in 2024 broadening the category of obesity to include individuals whose BMI is between 25 and 29.9 kg/m^2^, classically considered in the overweight range, if WHtR is ≥0.5 and medical, functional, or psychological impairment are present.^31^ In 2025, the *Lancet Committee* recommended excess body adiposity to be confirmed with at least two anthropometric measures except when BMI is >40 kg/m^2^.^32^

Based on the data above, it is reasonable to speculate that measures of abdominal obesity could be a better discriminator of atherosclerosis development and progression. However, WC alone does not take into account that taller individuals tend to have higher values of WC compared to short counterparts.^33^ Therefore, WHtR is an attractive alternative as an anthropometric measure of obesity.

The coronary artery calcium (CAC) score is a well characterized marker of cardiovascular risk and a surrogate marker of coronary atherosclerosis in asymptomatic individuals.^34^^,^^35^ Association between CAC progression and atherosclerotic cardiovascular disease (ASCVD) has been previously described in multiple cohorts.^36^^,^^37^ Moreover, a CAC score of zero is protective for ASCVD compared to a CAC higher than zero,^38^ and evolving from a CAC of zero to a positive score is interpreted as part of the process of developing atherosclerosis.^39^

We analyzed the ELSA-Brasil cohort individuals who underwent repeated CAC assessment and had no calcification at baseline to determine whether WHtR overperforms WC and BMI in predicting future development of coronary calcification (CAC incidence). Additionally, we performed a detailed analysis in individuals who do not meet the current most widely used definition of obesity based on BMI ≥30 kg/m^2^ to evaluate how WC and WHtR perform in predicting the risk of incident coronary atherosclerosis in this subgroup.

## Methods

### Sample

The Brazilian Longitudinal Study of Adult Health (ELSA-Brasil) design and goals have been previously published.^40^^,^^41^ In short, this racially mixed cohort recruited 15,105 volunteers aged 35–74 years, all active or retired employees from 6 education and research institutions in different Brazilian cities.^40^^,^^41^ Enrollment and baseline examination occurred between 2008 and 2010 and included medical interviews, clinical examinations, several biochemical analyses, and imaging studies.^41^ Individuals were followed up annually with telephone surveillance and had repeated study visits from 2012 to 2014 (second) and 2017 to 2018 (third).

Our study included all participants with no history of cardiovascular disease and two interval CAC assessments (first in 2010 through 2014 and a repeated scan in 2016 through 2018) with the first CAC being zero. All individuals with CAC assessments were from the São Paulo center.

### Data collection

Social and demographic characteristics, medical history, medications, anthropometric data, and blood pressure at rest were collected during in-person interviews and clinical examinations. Sex was self-reported as female or male. Race was self-identified according to the categories used in the Brazilian Census: Black, Brown (Mixed), White, Asian, and Indigenous. Smoking/tobacco use status (never, former, or current smoker) was self-reported as past and current cigarette. The leisure-time international physical activity questionnaire was used to quantify the level of physical activity. The first visit was used as baseline for this study.

ELSA-Brasil was approved by the ethics committees of all participating institutions. STROBE reporting guidelines for cohort studies were followed (Supplementary Appendix).

### Anthropometric measures

Body weight was measured by an electronic scale (Toledo, model 2096PP) with a precision of 50 g and maximum weight of 200 kg. WC was measured with a tape at the midpoint between the iliac crest and the lower border of the lowest rib with arms crossed in front of the chest and clothes lifted at the measurement site. Height was measured by a wall stadiometer (Seca, Hamburg, BRD) with a precision of 1 mm, and individuals were instructed to touch their head, buttocks, and heels on the wall and stare in the horizontal plane. Individuals were fasting, barefoot, with empty bladder, and wearing a standardized uniform and underwear. BMI was calculated by dividing weight in kg by squared height in m^2^. WHtR was calculated by dividing WC in cm by height in cm.

### CAC assessment

Images were performed on a 64-slice scanner (Philips, Amsterdam, Netherlands Brilliance, Philips), with CAC scoring standard technique, including prospective acquisition in mid-diastole, 120 kVp tube voltage, and variable current based on BMI. CAC was assessed by the Agatston method using the Brilliance Workspace software. The repeated test was performed on the same scanner, using identical acquisition parameters and the same software version.

### Statistical analyses

Baseline characteristics were stratified by WHtR with a cutoff of 0.5. Categorical variables were compared between those with WHtR <0.5 and ≥0.5 using the chi-square test. Continuous variables with normal distribution were tested using Student's *t*-test. Triglycerides had a non-normal distribution and were tested with a Wilcoxon rank-sum test. Normally distributed variables were presented as mean ± standard deviation (SD), and non-normally distributed reported as median with interquartile range (IQR 25–75th).

Because of high collinearity, BMI, WC, and WHtR were not included simultaneously within the same regression model. Given the low unit of WHtR (<1) and lack of uniformity in the units between WHtR, BMI, and WC, we assessed these variables per standard deviation from the mean. We calculated the incidence of CAC by BMI, WC, and WHtR strata and compared the area under the curve (AUC) of the receiver-operating characteristics (ROC) for BMI, WC, and WHtR with CAC incidence as the outcome using DeLong test^42^ to compare the AUCs of the predictive models. We then performed logistic regression analyses of BMI, WC, and WHtR to determine the odds of incident CAC on follow-up. The multivariate regression analyses were initially adjusted for the interscan period and the non-modifiable factors age, sex, race and family history of early myocardial infarction (Model 1), further adjusted for behavioral factors such as smoking status and physical activity (Model 2), and finally adjusted for clinical and biochemical factors such as diabetes, hypertension, high-density lipoprotein cholesterol (HDL-c), low-density lipoprotein cholesterol (LDL-c), log-transformed triglycerides and statin use (Model 3). As triglycerides had a highly skewed distribution, analyses were performed after its logarithm transformation. Participants were excluded from the model if data for at least one of the variables were missing. Since statins affect coronary calcification, sensitivity analyses for individuals who were not on statin were performed.

Furthermore, we tested these models using binary classifications (WHtR <0.50 vs ≥0.50, WHtR <0.55 vs ≥0.55, BMI 20–24.9 vs 25–29.9 vs ≥30 kg/m^2^, and WC <80 vs ≥80 cm for females and <90 vs ≥90 cm for males). The literature contains different cutoffs for WC and WHtR. The decision to use a WC cutoff 80/90 cm was made based on the International Diabetes Federation recommendation for South American populations.^43^ Additionally, this is in the lower end of WC across the several established cutoffs, which aligns with the goal of testing it in individuals with a BMI <30 kg/m^2^. For WHtR, EASO has recently recommended a cutoff of 0.5 for individuals with BMI in the overweight range^31^ and a previous ELSA-Brasil study has shown optimal cutoff to be 0.55 for women and 0.54 for men in assessing metabolic syndrome,^28^ so the decision was made to test both 0.5 and 0.55 as cutoffs. We also compared the ROC AUC for BMI, WC and WHtR in predicting CAC incidence for those with BMI <30 kg/m^2^ vs ≥30 kg/m^2^.

To explore the mechanism behind WHtR and CAC incidence association, mediation analysis was performed with other components of the metabolic syndrome: systolic and diastolic blood pressure, fasting glucose, serum triglycerides (after log transformation due to skewness), and serum HDL-cholesterol. Structural Equation Modeling was carried out with WHtR as a predictor and other variables as mediators of CAC incidence. Mediators were entered simultaneously with the assumption of no unmeasured mediator-outcome confounders to assess the indirect coefficients of the mediators and the direct coefficient of WHtR. We then built a model with WHtR as the sole predictor of CAC incidence to find the total WHtR effect coefficient. The proportion mediated was calculated by using the formula (total effect − direct effect)/total effect.

All analyses were performed using Stata BE 18 and 19 (StataCorp, College Station, TX). A two-sided p < 0.05 was considered statistically significant.

### Role of the funding source

Funders had no role in study design, data collection, data analysis, interpretation, and writing of this manuscript.

## Results

### Participants

**Fig. 1:**
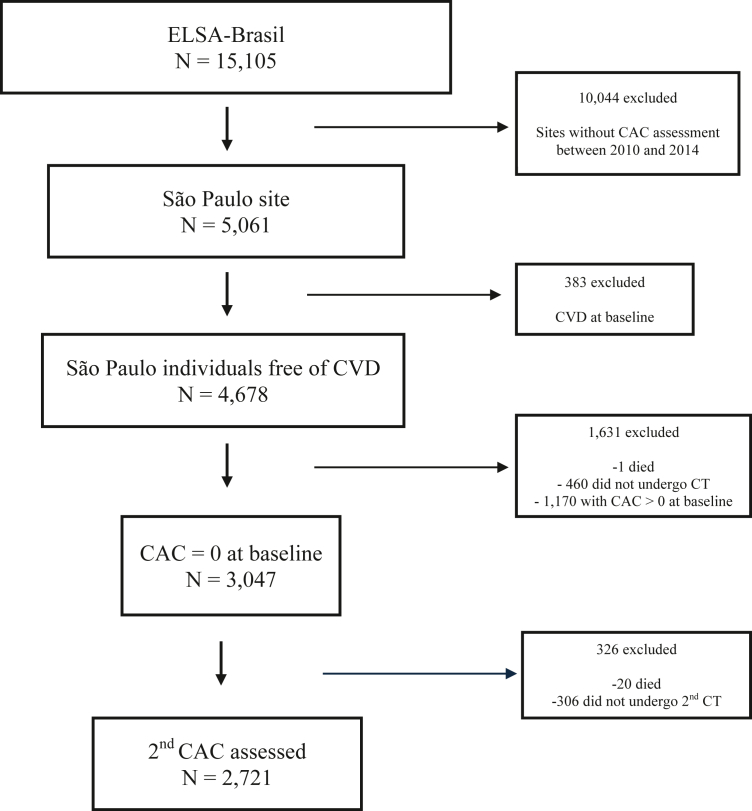
Participant flow diagram.

From 5061 participants in the São Paulo Center, 3047 had no cardiovascular disease at baseline and an initial CAC of zero. A total of 2721 had a repeated CAC assessment and were included in this study (Fig. 1). Individuals who underwent the first CAC assessment with a CAC score of zero but were excluded from this study due to the absence of the second CAC assessment were more often males, slightly older, with a minimal increased WC and WHtR. Baseline characteristics of these individuals, compared with those included in the study, are presented in the Supplementary Appendix.

### Baseline characteristics

Table 1 reports baseline characteristics of participants. Mean age was 48.1 ± 7.56 years and 62.6% were female. Most Individuals self-reported themselves as White, Brown (mixed), or Black: 57%, 22.8%, and 15.4%, respectively. The mean WHtR was 0.538 ± 0.073, 31.7% of individuals had a ratio lower than 0.5, while 68.3% had a ratio of at least 0.5. The mean BMI was 27.1 ± 4.88 kg/m^2^, and the mean waist circumference 88.2 ± 12.1 cm. Overall, 37% had a normal BMI (<25 kg/m^2^), 40.3% had a BMI between 25 and 29.9 kg/m^2^, and 22.7% had BMI in the obesity range (≥30 kg/m^2^). Those with higher WHtR were older, had a higher BMI, and were more likely to have diabetes, hypertension, and dyslipidemia. The mean interscan period was 5.24 ± 2.37 years (Table 1).

**Table 1:** Baseline characteristics.

	Waist-to-height ratio (WHtR)			
	Normal (<0.5)	Elevated (≥0.5)	Total	p-value
Number of individuals	863 (31.7%)	1858 (68.3%)	2721 (100%)	
Age	46.5 (7.34)	48.9 (7.54)	48.1 (7.56)	<0.0001
Sex				0.24
Male	309 (35.8%)	709 (38.2%)	1018 (37.4%)	
Female	554 (64.2%)	1149 (61.8%)	1703 (62.6%)	
Race				<0.001
Black	107 (12.5%)	309 (16.8%)	416 (15.4%)	
Brown/mixed	172 (20.1%)	441 (24%)	613 (22.8%)	
White	527 (61.5%)	1008 (54.9%)	1535 (57%)	
Asian	48 (5.6%)	59 (3.2%)	107 (4%)	
Native/Indigenous	3 (0.4%)	20 (1.1%)	23 (0.9%)	
Family history of early myocardial infarction	92 (10.7%)	230 (12.5%)	322 (11.9%)	0.18
Waist circumference (cm)	76.5 (6.19)	93.7 (10.2)	88.2 (12.1)	<0.0001
BMI (kg/m^2^)	22.7 (2.16)	29.1 (4.43)	27.1 (4.88)	<0.0001
Waist-to-height ratio	0.462 (0.029)	0.574 (0.059)	0.538 (0.073)	<0.0001
BMI categories (kg/m^2^)				<0.001
<25	746 (86.4%)	262 (14.1%)	1008 (37%)	
25–29.9	117 (13.6%)	980 (52.7%)	1097 (40.3%)	
30–34.9	0	429 (23.1%)	429 (15.8%)	
≥35	0	187 (10.1%)	187 (6.9%)	
Diabetes mellitus	49 (5.7%)	348 (18.7%)	397 (14.6%)	<0.001
HbA1C (%)	5.23 (0.599)	5.45 (0.881)	5.38 (0.809)	<0.0001
Hypertension	88 (10.2%)	536 (28.9%)	624 (22.9%)	<0.001
Systolic blood pressure (mmHg)	112 (13.6)	119 (14.7)	117 (14.8)	<0.0001
Diastolic blood pressure (mmHg)	70 (9.5)	76 (10)	74 (10.2)	<0.0001
Tobacco use				<0.001
Former	188 (21.8%)	580 (31.2%)	768 (28.2%)	
Current	127 (14.7%)	259 (13.9%)	386 (14.2%)	
Physical activity (including leisure)				<0.001
None	610 (73.6%)	1486 (83.2%)	2096 (80.2%)	
<150 min/week	125 (15.1%)	196 (11%)	321 (12.3%)	
≥150 min/week	94 (11.3%)	104 (5.8%)	198 (7.6%)	
Dyslipidemia	361 (41.8%)	1054 (56.7%)	1415 (52%)	<0.001
Total cholesterol (mg/dL)	203 (35.6)	214 (40.3)	211 (39.1)	<0.0001
Triglycerides (mg/dL)	85 [66–115]	120 [86–169]	107 [77–154]	<0.0001
HDL cholesterol (mg/dL)	61 (15.2)	55 (13.4)	57 (14.3)	<0.0001
LDL cholesterol (mg/dL)	123 (30.7)	132 (33.8)	129 (33.1)	<0.0001
Interscan period (years)	5.32 (0.905)	5.23 (2.8)	5.24 (2.37)	0.76

Continuous variables with normal distribution are presented as mean (standard deviation), continuous variables with non-normal distribution are presented as median [IQR], and categorical variables are presented as number (percentage).

### CAC incidence by BMI, WC, and WHtR

**Fig. 2:**
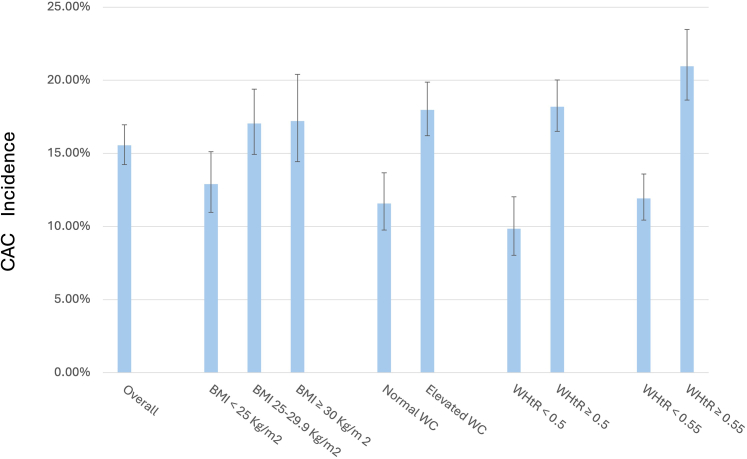
CAC incidence and 95% CI according to 3 different anthropometric measures of obesity.

CAC incidence was observed in 423 of 2721 participants (15.5% [CI 95%: 14.2–17%]) (Fig. 2). The incidence for those with BMI <25 kg/m^2^ was 12.9% (CI 95%: 11–15.1%), while for individuals with BMI between 25 and 29.9 kg/m^2^ it was 17% (CI 95%: 14.9–19.4%) and for those with BMI ≥30 kg/m^2^, 17.2% (CI 95%: 14.4–20.4%). The CAC incidence for individuals with WC lower than 80 cm for females or 90 cm for males was 11.6% (CI 95%: 9.7–13.7%), while for those with WC at or above this threshold, it was 18% (CI 95%: 16.2–19.9%). Incidence of CAC in those with WHtR lower than 0.5 was 9.8% (CI 95%: 8–12%) and for those with WHtR greater than or equal to 0.5 it was 18.2% (CI 95% 16.5–20%). CAC incidence in those with WHtR <0.55 was 11.9% (95% CI 10.4–13.6%) and for those with WHtR ≥0.55, it was 21% (95% CI 18.6–23.4%) (Fig. 2).

In predicting CAC incidence, the AUC of the ROC was 0.5652, 0.5925, and 0.6064 for BMI, WC, and WHtR respectively (Supplementary Appendix). The AUC of WC was larger than the AUC of the BMI (p: 0.001). The AUC of WHtR was significantly greater than the AUC of WC (p: 0.0384) and BMI (p < 0.0001).

In the logistic regression, there was an association between CAC incidence and BMI, WC, and WHtR in the crude analysis. The odds ratio (OR) for BMI was 1.19 (95% CI, 1.08–1.31) per 1 SD increase, while for WC it was 1.37 (95% CI, 1.23–1.52) per 1 SD increase and, for WHtR, 1.39 (95% CI, 1.25–1.54) per 1 SD increase. In the fully adjusted model, both WC and BMI were not associated with CAC incidence (p > 0.05) and WHtR was the only predictor of coronary calcification (OR per 1 SD increase, 1.18 [95% CI, 1.03–1.35]) (Table 2). As statins may accelerate coronary calcification, sensitivity analyses restricted to those not taking the medication were performed and yielded similar results with BMI having an OR of 1.13 (CI 95% 0.99–1.3), WC 1.16 (CI 95%: 0.99–1.35), and WHtR 1.24 (CI 95%: 1.07–1.44) for CAC incidence in the fully adjusted model (Supplementary Appendix). Height was not associated with CAC incidence (OR 0.99, CI 95%: 0.98–1.0, p: 0.3). Mediation analysis with WHtR as a predictor and markers of metabolic syndrome, such as systolic blood pressure, diastolic blood pressure, serum glucose, logarithm of serum triglycerides, and serum HDL cholesterol as mediators of CAC incidence, revealed that WHtR has a partial independent effect on CAC incidence (p: 0.034) with 63% of the effect being mediated (Supplementary Appendix).

**Table 2:** Logistic regression analysis for predictors of CAC incidence according to WHtR, WC and BMI as continuous standardized variables per 1 SD.

Standardized variable	Crude	Model 1	Model 2	Model 3
All individuals—n = 2721				
Body mass index	1.19 (1.08–1.31)^a^ N = 2721	1.22 (1.1–1.35)^a^ N = 2669	1.26 (1.13–1.4)^a^ N = 2565	1.08 (0.96–1.22) N = 2512
Waist circumference	1.37 (1.23–1.52)^a^ N = 2721	1.3 (1.15–1.46)^a^ N = 2669	1.33 (1.18–1.5)^a^ N = 2565	1.12 (0.98–1.29) N = 2512
Waist-to-height ratio	1.39 (1.25–1.54)^a^ N = 2721	1.34 (1.2–1.5)^a^ N = 2669	1.38 (1.23–1.55)^a^ N = 2565	1.18 (1.03–1.35)^a^ N = 2512
BMI <25 kg/m^2^—n = 1008				
Waist circumference	2.66 (1.86–3.79)^a^ N = 1008	2.81 (1.82–4.36)^a^ N = 990	2.59 (1.65–4.05)^a^ N = 947	1.94 (1.19–3.18)^a^ N = 935
Waist-to-height ratio	3.86 (2.56–5.83)^a^ N = 1008	3.1 (2.01–4.79)^a^ N = 990	2.84 (1.82–4.43)^a^ N = 947	2.1 (1.29–3.43)^a^ N = 935
BMI 25–29.9 kg/m^2^—n = 1097				
Waist circumference	1.79 (1.34–2.4)^a^ N = 1097	1.42 (0.99–2.05) N = 1070	1.48 (1.01–2.15)^a^ N = 1025	1.29 (0.86–1.92) N = 1002
Waist-to-height ratio	2.5 (1.77–3.52)^a^ N = 1097	1.78 (1.22–2.59)^a^ N = 1070	1.88 (1.28–2.76)^a^ N = 1025	1.7 (1.13–2.55)^a^ N = 1002
BMI ≥30 kg/m^2^—n = 616				
Waist circumference	1.25 (0.96–1.64) N = 616	1.11 (0.82–1.52) N = 604	1.15 (0.84–1.58) N = 588	0.99 (0.7–1.41) N = 571
Waist-to-height ratio	1.26 (0.96–1.65) N = 616	1.26 (0.95–1.69) N = 604	1.32 (0.98–1.78) N = 588	1.12 (0.8–1.57) N = 571

Model 1: adjusted for age, sex, race, family history of early ASCVD, and interscan period; Model 2: Model 1 plus smoking status and physical activity; Model 3: Model 2 plus diabetes, hypertension, HDL-c, LDL-c, log-transformed triglycerides, and statin use at baseline.

a Means p < 0.05.

When stratified by BMI, both WHtR and WC were predictors of CAC incidence for those with BMI <25 kg/m^2^ in all models, but for those with BMI in the 25–29.9 kg/m^2^ range, only WHtR was a predictor in the fully adjusted model (Table 2). WHtR and WC were not associated with CAC incidence in those with BMI >30 kg/m^2^ (Table 2). In predicting CAC incidence in those with BMI <30 kg/m^2^, the WHtR AUC slightly overperformed WC (0.635 vs 0.615 respectively, p: 0.046). For those with BMI >30 kg/m^2^, the AUC was 0.571 for WHtR and 0.541 for WC (p: 0.19) (Supplementary Appendix).

Stratification of WHtR, BMI, and WC in categorical variables showed that the increased level of classification for all markers was associated with higher OR for CAC in the follow-up scan in the crude models (Table 3). Compared to BMI <25 kg/m^2^, the OR for a BMI between 25 and 29.9 kg/m^2^ was 1.39 (95% CI, 1.0.9–1.77) and for a BMI ≥30 kg/m^2^, 1.4 (95% CI, 1.06–1.85). Those with WC greater than or equal to 80 cm in females or 90 cm in males had an OR of 1.67 (95% CI, 1.33–2.1) compared to individuals with a WC below this threshold. The OR for WHtR was 2.04 (95% CI, 1.58–2.62) and 1.96 (95% CI, 1.59–2.41) for a cutoff of 0.5 and 0.55 respectively. In the fully adjusted model, WHtR cutoff of 0.5 and 0.55 and WC cutoff 80/90 cm were predictors of coronary calcification (Table 3).

**Table 3:** Logistic regression analysis for predictors of CAC incidence by different categorized anthropometric measures in the entire sample and in those with BMI <30 kg/m^2^.

	Crude	Model 1	Model 2	Model 3
All individuals—n = 2721				
BMI 25–29.9 kg/m^2^^b^	1.39 (1.09–1.77)^a^ N = 2105	1.38 (1.07–1.78)^a^ N = 2064	1.4 (1.08–1.83)^a^ N = 1976	1.17 (0.88–1.55) N = 1941
BMI ≥30 kg/m^2^^b^	1.4 (1.06–1.85)^a^ N = 1624	1.41 (1.05–1.9)^a^ N = 1599	1.54 (1.13–2.09)^a^ N = 1540	1.03 (0.72–1.47) N = 1510
WC (F:80 cm/M:90 cm)	1.67 (1.33–2.1)^a^ N = 2721	1.72 (1.35–2.18)^a^ N = 2669	1.74 (1.36–2.24)^a^ N = 2565	1.36 (1.03–1.79)^a^ N = 2512
WHtR 0.50 cutoff	2.04 (1.58–2.62)^a^ N = 2721	1.81 (1.39–2.36)^a^ N = 2669	1.85 (1.41–2.43)^a^ N = 2565	1.4 (1.04–1.89)^a^ N = 2512
WHtR 0.55 cutoff	1.96 (1.59–2.41)^a^ N = 2721	1.78 (1.43–2.22)^a^ N = 2669	1.86 (1.48–2.34)^a^ N = 2565	1.48 (1.16–1.9)^a^ N = 2512
BMI <25 kg/m^2^—n = 1008				
WC (F:80 cm/M:90 cm)	2.25 (1.49–3.4)^a^ N = 1008	2.28 (1.46–3.56)^a^ N = 990	1.97 (1.24–3.13)^a^ N = 947	1.58 (0.96–2.59) N = 935
WHtR 0.50 cutoff	2.29 (1.56–3.35)^a^ N = 1008	1.9 (1.27–2.85)^a^ N = 990	1.79 (1.18–2.72)^a^ N = 947	1.39 (0.88–2.19) N = 935
BMI 25–29.9 kg/m^2^—n = 1097				
WC (F:80 cm/M:90 cm)	1.3 (0.84–1.99) N = 1097	1.24 (0.78–1.98) N = 1070	1.34 (0.82–2.18) N = 1025	1.24 (0.74–2.07) N = 1002
WHtR 0.50 cutoff	2.66 (1.32–5.36)^a^ N = 1097	1.9 (0.92–3.9) N = 1070	1.89 (0.91–3.92) N = 1025	1.77 (0.81–3.85) N = 1002
WHtR 0.55 cutoff	2.28 (1.65–3.15)^a^ N = 1097	1.77 (1.26–2.51)^a^ N = 1070	1.88 (1.31–2.68)^a^ N = 1025	1.77 (1.22–2.56)^a^ N = 1002

Model 1: adjusted for age, sex, race, family history of early ASCVD, and interscan period; Model 2: Model 1 plus smoking status and physical activity; Model 3: Model 2 plus diabetes, hypertension, HDL-c, LDL-c, log-transformed triglycerides, and statin use at baseline.

a Means p < 0.05.

b Compared to BMI <25 kg/m^2^.

### WC and WHtR as predictors of CAC incidence in individuals with BMI <30 kg/m^2^

All 616 individuals with BMI ≥30 kg/m^2^ had a WHtR ≥0.5 and 614 of them had a WC of at least 80 cm if female or 90 cm if male. We performed further analysis excluding the population with BMI ≥30 kg/m^2^ and categorizing their WHtR with a cutoff of either 0.5 or 0.55 and a WC cutoff of 80 cm for females and 90 cm (Table 3).

Only 19 of the 1008 individuals with BMI <25 kg/m^2^ had a WHtR ≥0.55, limiting the feasibility of further analysis. In this population with BMI <25 kg/m^2^, a WC cutoff of 80 cm for females or 90 cm for males and a WHtR cutoff of 0.5 were associated with CAC incidence with an OR of 2.25 (95% CI 1.49–3.4) and 2.29 (95% CI: 1.56–3.35), respectively. Using these cutoffs, both anthropometric measures were no longer associated with incident CAC in the fully adjusted model (Table 3).

In those with BMI between 25 and 29.9 kg/m^2^, WHtR ≥0.5 and ≥0.55 were associated with incident coronary calcification with an OR of 2.66 (95% CI 1.32–5.36) and 2.28 (95% CI 1.65–3.15), respectively, in the crude analysis. In this subset of the population within the overweight BMI range, a WC cutoff of 80 cm for females and 90 cm for males was not a predictor of CAC incidence even in the crude analysis, OR 1.3 (95% CI, 0.84–1.99), while WHtR with a cutoff of 0.5 was a predictor only in the crude model. A cutoff of 0.55 was significantly associated with future coronary calcification development even in the fully adjusted model (OR 1.77 [95% CI, 1.22–2.56]) (Table 3).

**Fig. 3:**
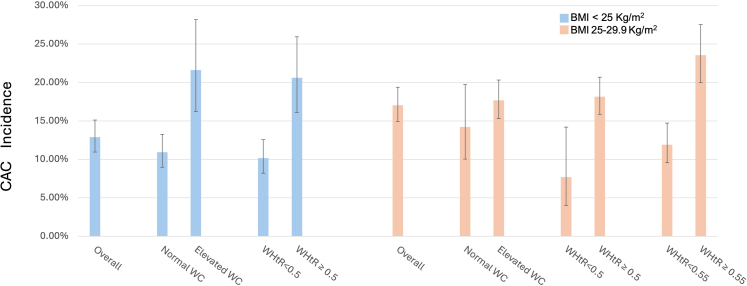
CAC incidence according to WC and WHtR in individuals with BMI < 30 kg/m^2^.

For individuals with a BMI <25 kg/m^2^ and WC <80 cm for females or <90 cm for males, the CAC incidence was 10.9% (CI 95%: 9–13.3%), while it was 21.6% (CI 95%: 16.2–28.2%) for those with WC ≥80 cm for females or ≥90 cm for males (Fig. 3). In the same population, the CAC incidence was 10.2% (CI 95%: 8.2–12.6%) in individuals with a WHtR <0.5 and 20.6% (CI 95%: 16.1–26%) in those with WHtR ≥0.5 (Fig. 3). Again, only 19 of the 1008 individuals with BMI <25 kg/m^2^ had a WHtR ≥0.55, limiting meaningful analysis for this population subset.

CAC incidence was 14.2% (CI 95%: 10–19.7%) in individuals with a BMI between 25 and 29.9 kg/m^2^ and WC <80 cm for females or <90 cm for females, and 17.7% (CI 95%: 15.3–20.3%) for those with WC ≥80 cm for females or ≥90 cm for males. Regarding WHtR in those with BMI within the overweight range, the CAC incidence was 7.7% (CI 95%, 4–14.2%) for those with a WHtR <0.5, and 18.2% (CI 95%: 15.9–20.7%) for those with WHtR ≥0.5, while for WHtR <0.55, the CAC incidence was 11.9% (CI 95% 9.6–14.7%), and 23.6% (CI 95% 20–27.6%) for WHtR ≥0.55 (Fig. 3).

## Discussion

This study demonstrated that WHtR is an independent predictor of CAC incidence, a proxy of the development of coronary atherosclerosis. While BMI and WC were associated with incident CAC in the unadjusted analysis, only WHtR remained a predictor in the fully adjusted model. Interestingly, this effect is essentially derived from individuals currently considered below the classical threshold for obesity (BMI <30 kg/m^2^). In particular, WHtR was a robust and independent predictor of CAC incidence in those with BMI in the overweight range (25 and 29.9 kg/m^2^), whereas WHtR and WC predicted incident CAC in individuals with normal BMI (<25 kg/m^2^).

The association between different anthropometric measures of obesity and prevalence of coronary calcification is well established. BMI was associated with CAC in a cross-sectional analysis of individuals in the USA.^2^ While one cross-sectional analysis from Korea found no association between BMI and WC with CAC score after adjustment for age and sex,^3^ another one showed BMI to be associated with severe calcification in those without diabetes^4^ and a third study found that increased BMI and WC were associated with CAC even after adjustment for confounders, with WC overperforming BMI.^5^ Cross-sectional non-adjusted studies assessing coronary stenosis by coronary angiography showed that WHtR was associated with coronary artery disease,^6^ and this association was stronger than that of BMI.^7^^,^^8^ WC was also associated with CAC density in a cross-sectional analysis of South Asian individuals who live in the USA after adjustment for confounders.^9^ Similarly, our group showed in a cross-sectional analysis that WHtR is associated with CAC score after adjustment for several potential confounders.^10^

It is also known that anthropometric obesity measures are associated with cardiovascular events and mortality. WC and WHtR were associated with cardiovascular mortality in the large Nurse Health Study cohort after adjustment for confounders without accounting for diabetes, hypertension and dyslipidemia.^11^ One analysis from the UK Biobank cohort has shown that BMI at the overweight and obesity range was associated with atherosclerotic cardiovascular disease in those without diabetes, and cardiovascular mortality irrespective of diabetes status.^12^ Another cohort from the UK Biobank showed that WHtR was associated with ischemic stroke and myocardial infarction after adjustment for multiple confounders.^13^ In a large study combining the Physician's Health Study and Women's Health Study cohorts, WHtR showed stronger association with a composite outcome of myocardial infarction, stroke, and cardiovascular death than BMI, even after adjustment for numerous confounders, including diabetes, dyslipidemia, and hypertension.^14^ Smaller cohorts with targeted populations showed that WHtR might outperform BMI and WC in predicting clinical cardiovascular outcomes in individuals with hypertension^15^ and diabetes.^16^^,^^17^

To the best of our knowledge this is the first longitudinal study showing that WHtR is independently associated with CAC incidence, even after adjustment for all classic cardiovascular risk factors, in a large, diverse population, particularly in those with BMI <30 kg/m^2^. Also relevant, mediation analysis with other components of metabolic syndrome showed a proportion mediated of 67%, suggesting that while some of the association between WHtR and CAC incidence is accounted for by other components of the metabolic syndrome, a significant portion is still attributed to WHtR independently of traditional risk factors. These findings support the idea that central obesity is associated with atherosclerosis development beyond classic cardiovascular risk factors. In clinical practice, compared to WC, WHtR also has additional advantages, such as not requiring different cutoffs based on sex and not overestimating central fat in taller individuals, who are expected to have increased WC.^44^

Although BMI alone does not discriminate body composition and distribution,^19^^,^^20^ the best way to move beyond it is still controversial. Since anthropometric measures of obesity are highly correlated, the improvement in the AUC of the ROC in this study was only marginal. Nevertheless, we have demonstrated that individuals with BMI in the overweight range and increased WHtR have higher incidence of coronary calcification. In this respect, further studies with larger populations and hard outcomes should be considered to assess if WHtR improves clinical cardiovascular risk prediction when compared to BMI, which is used in the PREVENT equation,^18^ particularly in those with BMI <30 kg/m^2^. Our findings also reinforce the EASO recommendation to broaden WHtR use in clinical practice to individuals with BMI between 25 and 29.9 kg/m^2^.^31^ Recently, glucagon-like peptide-1 receptor agonist treatment of individuals with BMI ≥27 kg/m^2^ showed to be beneficial in reducing cardiovascular and renal outcomes.^45^^,^^46^ The BMI cutoff for these trials does not correspond to the conventional threshold of 30 kg/m^2^, which is itself arbitrary, but instead reflects an alternative threshold without a strong basis. Additional research is required to assess the potential of WHtR in refining the concept of obesity.

Strengths of this study include the longitudinal prospective design, assessment of a non-referred multiracial diverse population with baseline data extracted years before the first CT, reducing the risk of reverse causation, and a long interscan period. It is important to highlight, however, that anthropometric measures (BMI, WC, and WHtR) are dynamic and changes over time were not accounted in our analyses, which considered values at the beginning of the study. Additionally, as multiple exploratory analyses were performed, type I error risk is increased. Although our results are consistent, validation of our findings in different cohorts is appropriate. It is also necessary to point out that we have assessed CAC incidence as a marker of subclinical development of atherosclerosis, which is different than CAC progression in individuals with previous scores superior to zero. The scope of this study was to better understand how anthropometric measures associate with atherosclerosis development. Thus, the focus was understanding the link between anthropometric measures and atherosclerotic process rather than performing cardiovascular risk stratification, which would require a larger population with longer follow-up.

In conclusion, WHtR was the only anthropometric obesity measure that independently predicted future coronary artery calcification. This finding was driven by individuals with BMI <30 kg/m^2^, a group that is not considered with obesity by classical BMI criteria. A WHtR cutoff of 0.5 can predict future coronary calcification in individuals with BMI below the obesity range and is easily translated into clinical practice. However, a cutoff of 0.55 had a stronger and independent association with CAC incidence in individuals with BMI between 25 and 29.9 kg/m^2^ in this multiracial diverse population. Despite testing 0.5 and 0.55 as the classic WHtR cutoffs, the goal of this study was to compare WHtR with WC and BMI in predicting future development of coronary calcification, and so optimal WHtR cutoffs were not investigated.

## Contributors

TBM: conceptualization, methods, statistical analysis, writing, review, and editing of drafts.

GG: methods, statistical analysis, writing, review, and editing of drafts.

RCF: conceptualization, methods, review, and editing of drafts.

BH, CCPSJ, CMR, RDS, IB, PAL: conceptualization, review, and editing of drafts.

MSB: supervision of conceptualization, method, statistical analysis, writing, review and editing of drafts.

All authors have access to all data reported in the study.

## Data sharing statement

Data supporting the findings of this study are available upon reasonable request.

## Declaration of generative AI and AI-assisted technologies in the manuscript preparation process

During the preparation of this work the authors used ChatGPT in order to check grammar and improve the readability of a few sentences. After using this tool, the authors reviewed and edited the content as needed and take full responsibility for the content of the published article.

## Declaration of interests

Marcio S. Bittencourt has received speaker fees from Cleerly and consulting fees from Elucid.

Bruno Halpern has received honoraria related to consult, research, and speaker activities from Novo Nordisk, Eli-Lilly, Astra Zeneca, Boehringer Ingelheim, and Merck/Currax.

Raul D. Santos has received honoraria related to consulting, research, and speaker activities from Amgen, Amryt, Daiichi-Sankyo, Esperion, Eli-Lilly, Kowa, Libbs, Novo-Nordisk, Novartis, PTC Therapeutics, Torrent and Sanofi/Regeneron.

All the other authors have no disclosures.
