# Supplementary material for: Waist-to-height ratio and coronary artery calcium incidence: the Brazilian Longitudinal Study of Adult Health (ELSA-Brasil)
Source: Lancet Reg Health Am. 2025 Oct 31;52:101281. doi: 10.1016/j.lana.2025.101281 (PMC12800998; doi:10.1016/j.lana.2025.101281)
Supplement: Supplementary Tables and Figures [file mmc1.docx]

**Caption for supplementary material**

Table 1S. Baseline characteristics of individuals who had the 1^st^ CAC assessment and did not undergo the 2^nd^ CT.

Table 2S. Logistic regression analysis for predictors of CAC incidence according to WHtR, WC and BMI as continuous standardized variables per 1 SD in individuals not taking statin (fully adjusted)

Table 3S and Flow chart. Structural Equation Modeling: WHtR as a predictor and systolic blood pressure, diastolic blood pressure, serum fasting glucose, serum HDL cholesterol, and triglycerides logarithm as mediators of CAC incidence.

Table 4S and Flow chart. Structural Equation Modeling: WHtR as a predictor of CAC incidence with proportion of WHtR mediation.

Figure 1S. ROC curve evaluating the predictive accuracy of BMI for incident CAC

Figure 2S. ROC curve evaluating the predictive accuracy of WC for incident CAC

Figure 3S. ROC curve evaluating the predictive accuracy of WHtR for incident CAC

Figure 4S. ROC curve evaluating the predictive accuracy of WHtR for incident CAC in individuals with BMI < 30 Kg/m^2^

Figure 5S. ROC curve evaluating the predictive accuracy of WC for incident CAC in individuals with BMI < 30 Kg/m^2^

Figure 6S. ROC curve evaluating the predictive accuracy of WHtR for incident CAC in individuals with BMI ≥ 30 Kg/m^2^

Figure 7S. ROC curve evaluating the predictive accuracy of WC for incident CAC in individuals with BMI ≥ 30 Kg/m^2^

Table 5S. STROBE checklist for cohorts.

Table 1S. Baseline characteristics of individuals who had the 1^st^ CAC=0 and underwent the 2^nd^ CT compared to those with a 1^st^ CAC=0 who did not undergo the 2^nd^ CT

|  | **Completed both CTs** | | **Missed the ^2nd^ CT** |  | **p-value** | |
| --- | --- | --- | --- | --- | --- | --- |
| **Number of individuals** | 2,721 | | 326 |  |  | |
| **Age** | 48.1 (7.56) | | 50.6 (8.25) |  | <0.0001 | |
| **Gender** |  | |  |  | 0.004 | |
| Male | 1,018 (37.4%) | | 149 (45.7%) |  |  | |
| Female | 1,703 (62.6%) | | 177 (54.3%) |  |  | |
| **Race** |  | |  |  | 0.36 | |
| Black | 416 (15.4%) | | 42 (13.0%) |  |  | |
| Brown/mixed | 613 (22.8%) | | 70 (21.7%) |  |  | |
| White | 1,535 (57%) | | 198 (61.5%) |  |  | |
| Asian | 107 (4%) | | 8 (2.5%) |  |  | |
| Native/indigenous | 23 (0.9%) | | 4 (1.2%) |  |  | |
| **Family history of early myocardial infarction** | 322 (11.9%) | | 43 (13.3%) |  | 0.8 | |
| **Waist circumference (cm)** | 88.2 (12.1) | | 89.9 (13) |  | 0.02 | |
| **BMI (kg/m^2^)** | 27.1 (4.88) | | 27.4 (5.06) |  | 0.23 | |
| **Waist-to-height ratio** | 0.538 (0.073) | | 0.548 (0.077) |  | 0.033 | |
| **BMI categories (kg/m^2^)** |  | |  |  | 0.13 | |
| < 25 | 1,008 (37%) | | 114 (35.0%) |  |  | |
| 25-29.9 | 1,097 (40.3%) | | 120 (36.8%) |  |  | |
| 30-34.9 | 429 (15.8%) | | 67 (20.6%) |  |  | |
| ≥ 35 | 187 (6.9%) | | 25 (7.7%) |  |  | |
| **Diabetes mellitus** | 397 (14.6%) | | 51 (15.6%) |  | 0.26 | |
| **HbA1C (%)** | 5.38 (0.81) | | 5.4(0.962) |  | 0.7 | |
| **Hypertension** | 624 (22.9%) | | 85 (26.1%) |  | 0.2 | |
| **Systolic Blood Pressure (mmHg)** | 117 (14.8) | | 119 (17.7) |  | 0.002 | |
| **Diastolic Blood Pressure (mmHg)** | 74 (10.2) | | 76 (11.7) |  | 0.001 | |
| **Tobacco use** |  | |  |  | 0.17 | |
| Former | 768 (28.2%) | | 97 (29.8%) |  |  | |
| Current | 386 (14.2%) | | 57 (17.5%) |  |  | |
| **Physical activity(including leisure)** | | 0.65 | | | |  |
| None | 2,096 (80.2%) | | 247 (79.4%) |  |  | |
| < 150 min/week | 321 (12.3%) | | 36 (11.6%) |  |  | |
| ≥ 150 min/week | 198 (7.6%) | | 28 (9.0%) |  |  | |
| **Dyslipidemia** | 1,415 (52%) | | 169 (51.8%) |  | 0.96 | |
| **Total cholesterol (mg/dL)** | 211 (39.1) | | 211 (40.6) |  | 0.99 | |
| **Triglycerides (mg/dL)** | 107 [77 – 154] | | 115 [81 - 158] |  | 0.1 | |
| **HDL cholesterol (mg/dL)** | 57 (14.3) | | 57 (14.6) |  | 0.84 | |
| **LDL cholesterol (mg/dL)** | 129 (33.1) | | 128 (33) |  | 0.45 | |

Data presented as mean (standard deviation), median [interquartile range] and number (percentage)

Table 2S. Logistic regression analysis for predictors of CAC incidence according to WHtR, WC and BMI as continuous standardized variables per 1 SD (CI 95%) in individuals not taking statin (fully adjusted)

| Standardized variable | Fully adjusted model OR | P value |
| --- | --- | --- |
| Body mass index | 1.13 (0.99-1.3)  N=2,156 | 0.064 |
|  |  |  |
| Waist Circumference | 1.16 (0.99-1.35)  N=2,156 | 0.068 |
|  |  |  |
| Waist-to-height ratio | **1.24 (1.07-1.44)**  N=2,156 | 0.005 |

*Adjusted for age, sex, race, and family history of early ASCVD, smoking status, physical activity, diabetes, hypertension, HDL-c, LDL-c, and log-transformed triglycerides.*

Table 3S and Flow chart. Structural Equation Modeling: WHtR as a predictor and systolic blood pressure, diastolic blood pressure, serum fasting glucose, serum HDL cholesterol, and triglycerides logarithm as mediators of CAC incidence.

Systolic blood pressure

Diastolic blood pressure

WHtR

CAC incidence

Fasting glucose

Triglycerides logarithm

HDL cholesterol

|  | Coefficient | CI 95% | P value |
| --- | --- | --- | --- |
| Systolic blood pressure (mmHg) | 0.0033788 | 0.0017408, 0.0050168 | <0.001 |
| Diastolic blood pressure (mmHg) | -0.0023131 | -0.0047364, 0.0001102 | 0.061 |
| Fasting glucose (mg/dL) | 0.0023758 | 0.0017172, 0.0030344 | <0.001 |
| HDL cholesterol (mg/dL) | 0.000281 | -0.0007117, 0.0012738 | 0.58 |
| Triglycerides (log transformed, mg/dL) | 0.0507369 | 0.0211346, 0.0803392 | 0.001 |
| WHtR (per SD) | 0.0168128 | 0.0013071, 0.0323184 | 0.034 |

*All predictors shown were simultaneously included in the model as predictors of CAC incidence. Coefficients represent the****direct (partial) effect****of WHtR and indirect effects of mediators on CAC incidence. The effect of WHtR on CAC incidence is****adjusted****for mediators. Coefficients are standardized for comparability across predictors. As triglycerides have a skewed distribution, it was placed in the model after log transformation. As WHtR unit is < 1, it was placed in the model per SD.*

Table 4S and Flow chart. Structural Equation Modeling: WHtR as a predictor of CAC incidence with proportion of WHtR mediation.

CAC incidence

|  | Coefficient | CI 95% | P value |
| --- | --- | --- | --- |
| WHtR (per SD) | 0.0454573 | 0.0314552, 0.0594594 | <0.001 |

WHtR

*WHtR as the sole predictor of CAC incidence with standardized coefficient.*

Proportion of WHtR effect that is mediated = (0.0454573 - 0.0168128) / 0.0454573 = 63%

Figure 1S. ROC curve evaluating the predictive accuracy of BMI for incident CAC


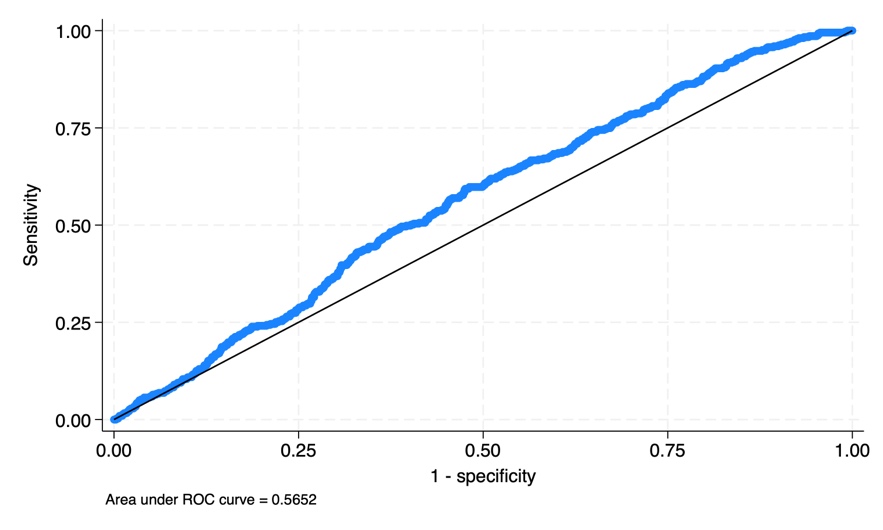


Figure 2S. ROC curve evaluating the predictive accuracy of WC for incident CAC


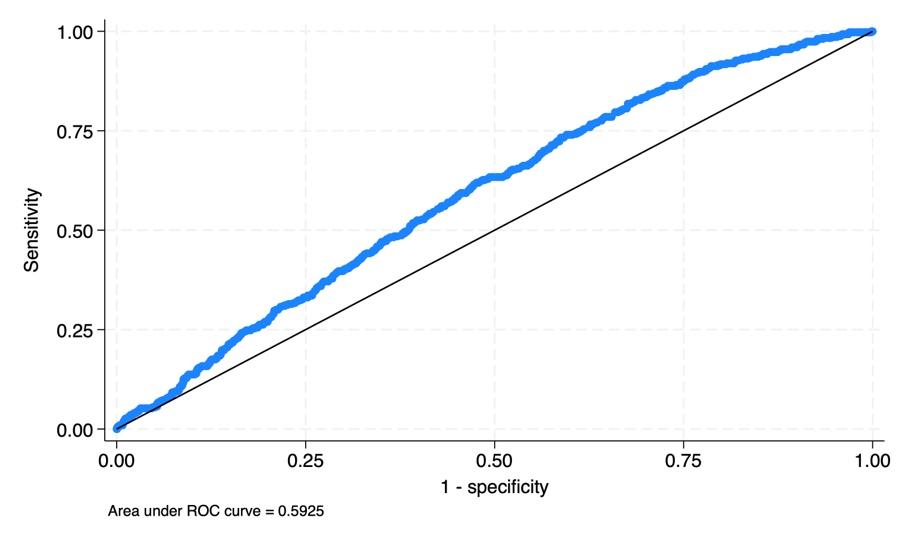


Figure 3S. ROC curve evaluating the predictive accuracy of WHtR for incident CAC


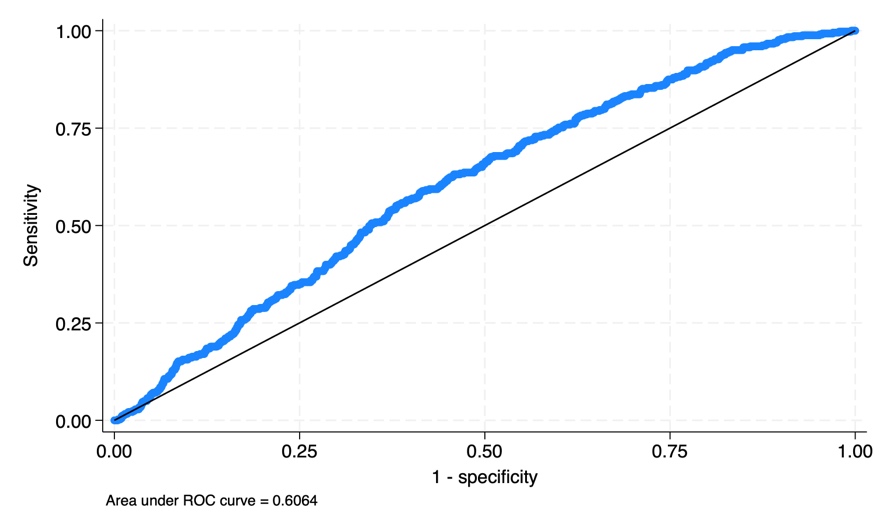


Figure 4S. ROC curve evaluating the predictive accuracy of WHtR for incident CAC in individuals with BMI < 30 Kg/m^2^


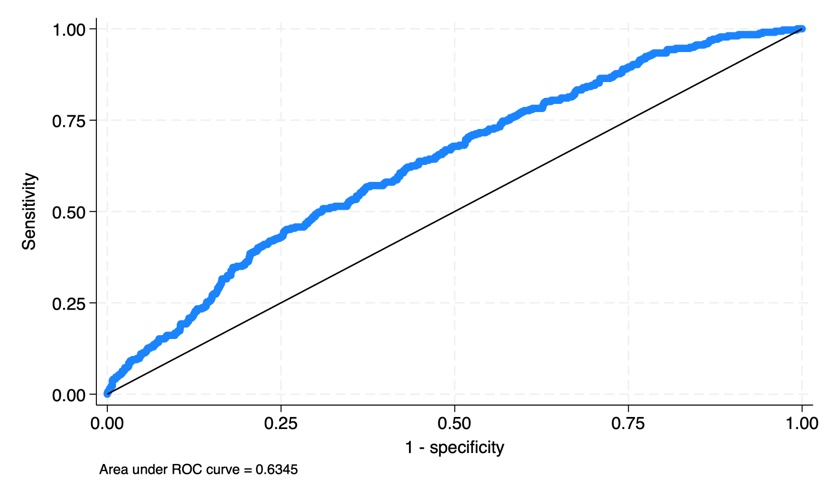


Figure 5S. ROC curve evaluating the predictive accuracy of WC for incident CAC in individuals with BMI < 30 Kg/m^2^

^
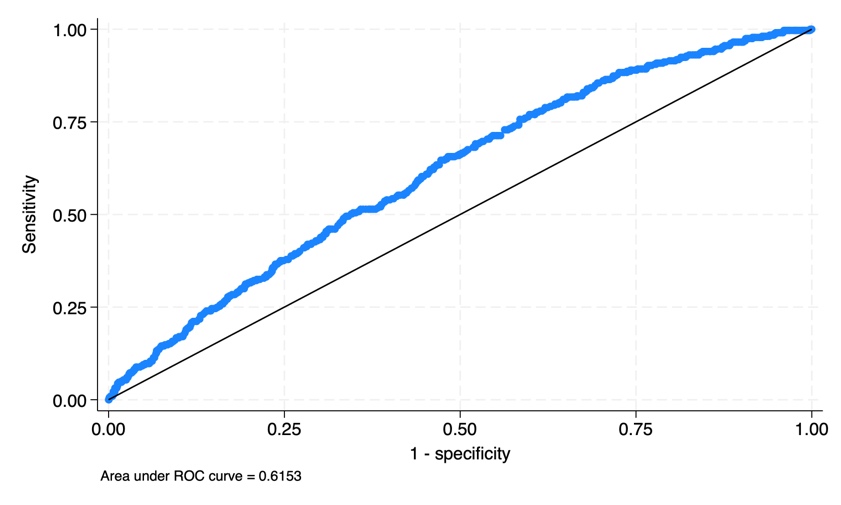
^

Figure 6S. ROC curve evaluating the predictive accuracy of WHtR for incident CAC in individuals with BMI ≥ 30 Kg/m^2^

^
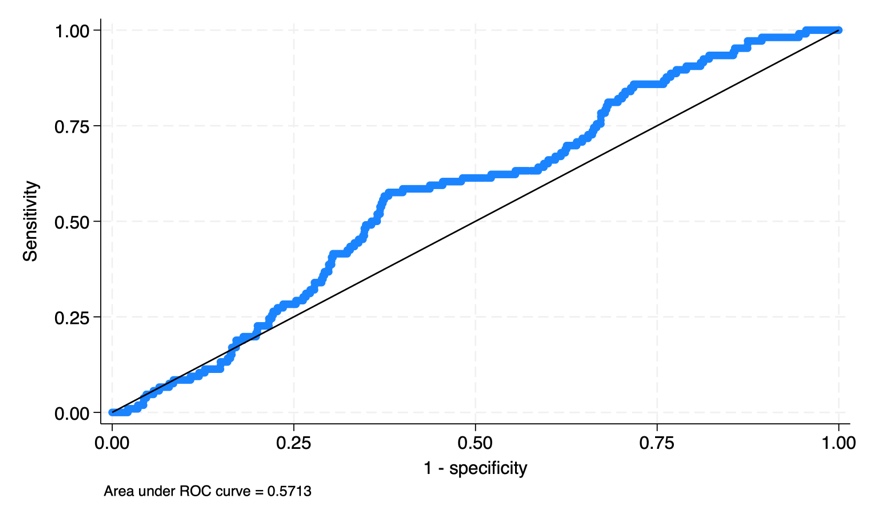
^

Figure 7S. ROC curve evaluating the predictive accuracy of WC for incident CAC in individuals with BMI ≥ 30 Kg/m^2^


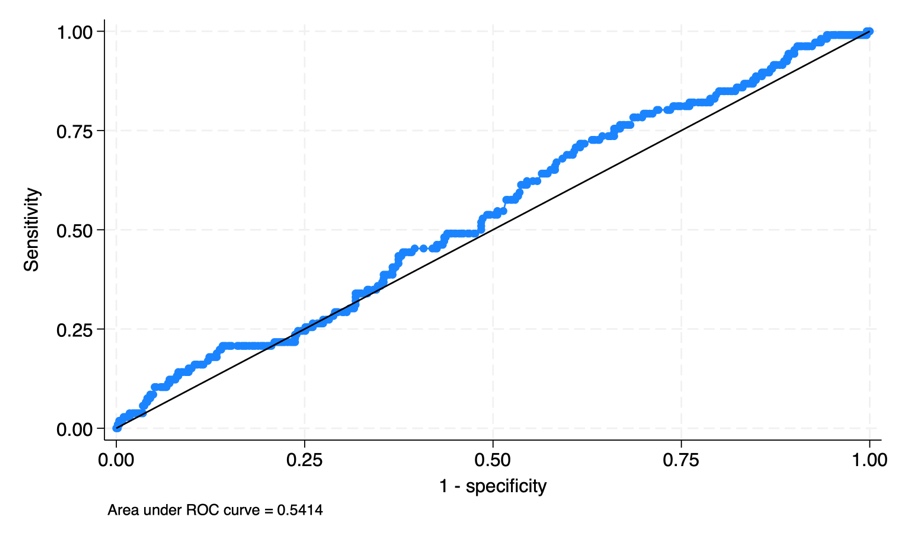


Table 5S. STROBE Statement (page number according to the submitted main manuscript)

|  | Item No | Recommendation | Page N^o^ |
| --- | --- | --- | --- |
| **Title and abstract** | 1 | (*a*) Indicate the study’s design with a commonly used term in the title or the abstract | Abstract |
|  |  | (*b*) Provide in the abstract an informative and balanced summary of what was done and what was found | Completed |
| Introduction | | | |
| Background/rationale | 2 | Explain the scientific background and rationale for the investigation being reported | 1-2 |
| Objectives | 3 | State specific objectives, including any prespecified hypotheses | 3 |
| Methods | | | |
| Study design | 4 | Present key elements of study design early in the paper | 3 |
| Setting | 5 | Describe the setting, locations, and relevant dates, including periods of recruitment, exposure, follow-up, and data collection | 3 |
| Participants | 6 | (*a*) Give the eligibility criteria, and the sources and methods of selection of participants. Describe methods of follow-up | (a) 3-4 |
|  |  | (*b*) For matched studies, give matching criteria and number of exposed and unexposed | (b) 3-4 |
| Variables | 7 | Clearly define all outcomes, exposures, predictors, potential confounders, and effect modifiers. Give diagnostic criteria, if applicable | 4-6 |
| Data sources/ measurement | 8* | For each variable of interest, give sources of data and details of methods of assessment (measurement). Describe comparability of assessment methods if there is more than one group | 4-5 |
| Bias | 9 | Describe any efforts to address potential sources of bias | 4-6 |
| Study size | 10 | Explain how the study size was arrived at | 3-7 (Figure 1) |
| Quantitative variables | 11 | Explain how quantitative variables were handled in the analyses. If applicable, describe which groupings were chosen and why | 4-6 |
| Statistical methods | 12 | (*a*) Describe all statistical methods, including those used to control for confounding | (a) 4-6 |
|  |  | (*b*) Describe any methods used to examine subgroups and interactions | (b) 4-6 |
|  |  | (*c*) Explain how missing data were addressed | (c) 3 |
|  |  | (*d*) If applicable, explain how loss to follow-up was addressed | (d) 3 |
|  |  | (*e*) Describe any sensitivity analyses | (e) 5 |
| Results | | |  |
| Participants | 13* | (a) Report numbers of individuals at each stage of study—eg numbers potentially eligible, examined for eligibility, confirmed eligible, included in the study, completing follow-up, and analysed | (a): 7 (figure 1)  (b): 7(figure 1) |
|  |  | (b) Give reasons for non-participation at each stage | (c) 7 (figure 1) |
|  |  | (c) Consider use of a flow diagram |  |
| Descriptive data | 14* | (a) Give characteristics of study participants (eg demographic, clinical, social) and information on exposures and potential confounders | (a): 7 (Table 1) |
|  |  | (b) Indicate number of participants with missing data for each variable of interest | (b): 8-10 (Tables 2- 3) |
|  |  | (c) Summarise follow-up time (eg, average and total amount) | (c) 7 (table 1) |
| Outcome data | 15* | Report numbers of outcome events or summary measures over time | 7-10 (figures 2-3) |
| Main results | 16 | (*a*) Give unadjusted estimates and, if applicable, confounder-adjusted estimates and their precision (eg, 95% confidence interval). Make clear which confounders were adjusted for and why they were included | (a) 7-10 (tables 2-3 and figures 2-3) |
|  |  | (*b*) Report category boundaries when continuous variables were categorized | (b) 7-10 (Tables2-3, figures 2-3) |
|  |  | (*c*) If relevant, consider translating estimates of relative risk into absolute risk for a meaningful time period | (c): N/A |
| Other analyses | 17 | Report other analyses done—eg analyses of subgroups and interactions, and sensitivity analyses | 7-10 (Tables 2-3, figures 2-3) |
| **Discussion** |  |  |  |
| Key results | 18 | Summarise key results with reference to study objectives | 11 |
| Limitations | 19 | Discuss limitations of the study, taking into account sources of potential bias or imprecision. Discuss both direction and magnitude of any potential bias | 13 |
| Interpretation | 20 | Give a cautious overall interpretation of results considering objectives, limitations, multiplicity of analyses, results from similar studies, and other relevant evidence | 11-13 |
| Generalisability | 21 | Discuss the generalisability (external validity) of the study results | 11-13 |
| **Other information** |  |  |  |
| Funding | 22 | Give the source of funding and the role of the funders for the present study and, if applicable, for the original study on which the present article is based | 6 |
